# Comparison of regmed and BayesNetty for exploring causal models with many variables

**DOI:** 10.1002/gepi.22532

**Published:** 2023-06-27

**Authors:** Richard Howey, Heather J. Cordell

**Affiliations:** ^1^ Population Health Sciences Institute Newcastle University Newcastle upon Tyne UK

**Keywords:** Bayesian networks, causal inference, mediation

## Abstract

Here we compare a recently proposed method and software package, regmed, with our own previously developed package, BayesNetty, designed to allow exploratory analysis of complex causal relationships between biological variables. We find that regmed generally has poorer recall but much better precision than BayesNetty. This is perhaps not too surprising as regmed is specifically designed for use with high‐dimensional data. BayesNetty is found to be more sensitive to the resulting multiple testing problem encountered in these circumstances. However, as regmed is not designed to handle missing data, its performance is severely affected when missing data is present, whereas the performance of BayesNetty is only slightly affected. The performance of regmed can be rescued in this situation by first using BayesNetty to impute the missing data, and then applying regmed to the resulting “filled‐in” data set.

## 1 MAIN TEXT

In a paper recently published in this journal, Schaid et al. (2022) describe a method and accompanying R package, regmed, for fitting penalized structural equation regression models to multivariate data. In their models, they restrict themselves to three types of prespecified variables—exposure variables, mediator variables, and outcome variables—and they are interested in detecting possible causal pathways between the different types of variables, in the specified order, while accounting for residual variances and covariances among the variables. Their method is designed to evaluate high‐dimensional data sets with many variables of each type.

In this report, we compare regmed with our Bayesian Network software, BayesNetty (Howey et al., 2020), using the same simulation models used by Schaid et al. (2022). We compute the network recall and precision of each method, varying the causal effect sizes and sample sizes (the recall is the percentage of “true” edges in the simulation model that are detected, and the precision is the percentage of detected edges that are indeed in the original simulation model). As one of BayesNetty's strengths is its ability to handle missing data through an imputation‐based approach (Howey et al., 2021), we also simulate variables with varying amounts of missing data and compare the two methods.

**Figure 1:**
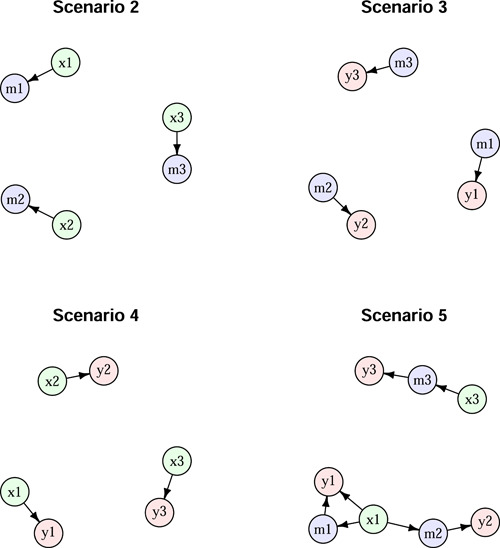
Simulation scenarios reproduced from Figure 2 of Schaid et al. (2022). Only variables with connecting edges are shown.

We simulated data as described in Schaid et al. (2022) under their scenarios 2–5 (see Figure 2 of Schaid et al., 2022) as shown here in Figure 1. The simulated data consisted of 80 exposure variables, 10 mediator variables, and 5 outcome variables; only those variables that were connected by causal relationships are shown in Figure 1. The data were simulated with multivariate normal distributions with each variable having mean 0, variance 1, and covariances assumed to be 0.5 between exposure variables, 0.2 between mediator variables, and 0.1 between outcome variables. Exposure variables were labeled $x_{1},x_{2}\ldots x_{80}$, mediator variables were labeled $m_{1},m_{2}\ldots m_{10}$ and outcome variables were labeled $y_{1},y_{2}\ldots y_{5}$. Sample sizes were set to 100, 500, and 1000 for a range of effect sizes corresponding to regression coefficients (0.1,0.2,…0.5) modeling the relationship between the variables joined by directed arrows.

We additionally simulated data under slightly different conditions. First by assuming lower covariances, set to 0.1 between exposure variables and also 0.1 between mediator variables, for scenarios 3 and 4. Second by using single nucleotide polymorphism (SNP) data (coded 0, 1, and 2) for the exposure variables instead of continuous values, for scenario 5. The SNPs were simulated using the R package echoseq (https://github.com/hruffieux/echoseq) assuming a Pearson correlation of 0.5.

Best‐fit networks were found using BayesNetty with the constraints that edges were only permitted from exposure variables to either mediator or outcome variables, and from mediator variables to outcome variables. This is equivalent to the constraints imposed by regmed when the variables are classed as being variables of a certain type.

We also simulated data sets with missing data where the mediator and outcome variables were set to be missing with a range of probabilities (0,0.05,…0.25). In this case, the effect size was set to 0.3. We used the option available in BayesNetty to impute the missing data before finding a best‐fit network, while for regmed it was necessary to first remove individuals with missing data from the data set before analysis. We also investigated outputting the imputed data set from BayesNetty for subsequent analysis with regmed.

In all simulation replicates, both regmed and BayesNetty were used to analyze the data and the recall and precision were calculated. All parameter settings for the simulations were repeated 100 times and average results are presented.

**Figure 2:**
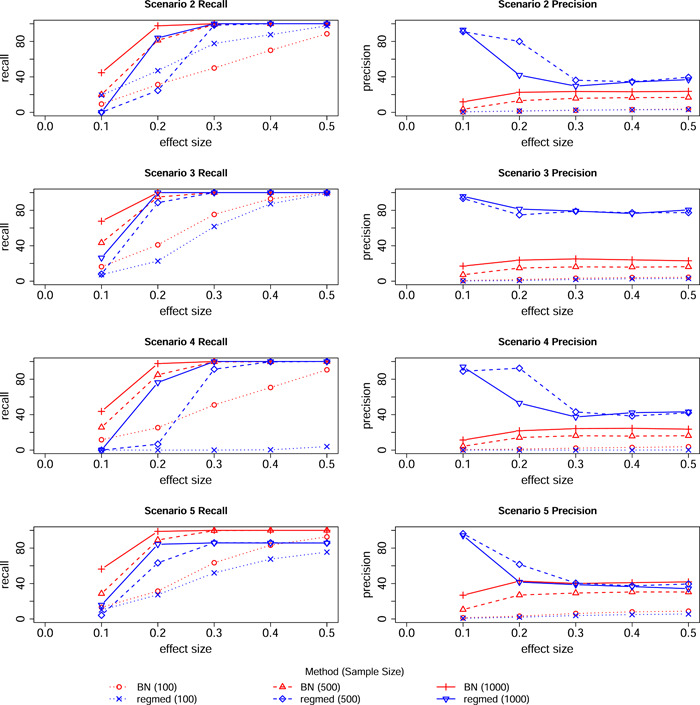
Network recall and precision. Results are for shown for the regmed and Bayesian network (BN) methods for varying values of the effect size for each scenario and sample sizes of 100, 500, and 1000.

Figure 2 shows the results for each parameter setting. BayesNetty shows better recall (i.e., better power to detect “true” edges) than regmed for every scenario and sample size considered. However, for sample size 500 or 1000, the precision for every scenario is better for regmed than BayesNetty (especially for low effect sizes), suggesting that BayesNetty's better power comes at the expense of a larger number of false positive edges. This is particularly evident in scenario 3 where there are only edges from mediators to outcome variables.

When the sample size is only 100, the precision is equally poor for each method. The only situation where BayesNetty outperforms regmed is under scenario 5 when the sample size is 1000 and the effect size is at least 0.3. Here BayesNetty shows better recall and BayesNetty's precision is around the same level as regmed.

**Figure 3:**
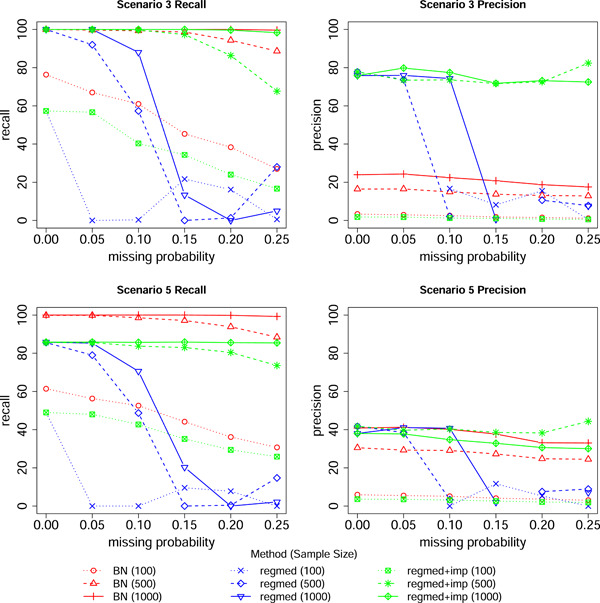
Network recall and precision with missing data. Results are shown for the regmed and Bayesian network (BN) methods for varying values of the probability of missing data for the mediation and outcome variables and sample sizes of 100, 500, and 1000. The effect size is fixed at 0.3 for each scenario. The option in BayesNetty to impute missing data before finding a best‐fit network was used for BN, while for regmed we considered both the default approach of a “complete case analysis” (where individuals with any missing data are removed) and also the option (regmed + imp) of outputting the imputed data set from BayesNetty for subsequent analysis in regmed.

Figure 3 shows the results when there is missing data, as the amount of missing data is increased. When the probability of missing data increases, the sample size for regmed effectively decreases, and so its recall dramatically falls. For BayesNetty this is not the case as the data is imputed before fitting a best‐fit network, and so we see only small drops in the recall and precision. The precision for regmed is seen to be quite erratic as the probability of missing data increases, as there are more simulated replicates with no edges detected, and so fewer valid findings from which to calculate an average precision. However, if the imputed data from BayesNetty is used as input to regmed, we see that the performance of regmed can be rescued and, similar to the results shown in Figure 2, regmed shows slightly lower levels of recall but considerably higher levels of precision than BayesNetty.

**Figure 4:**
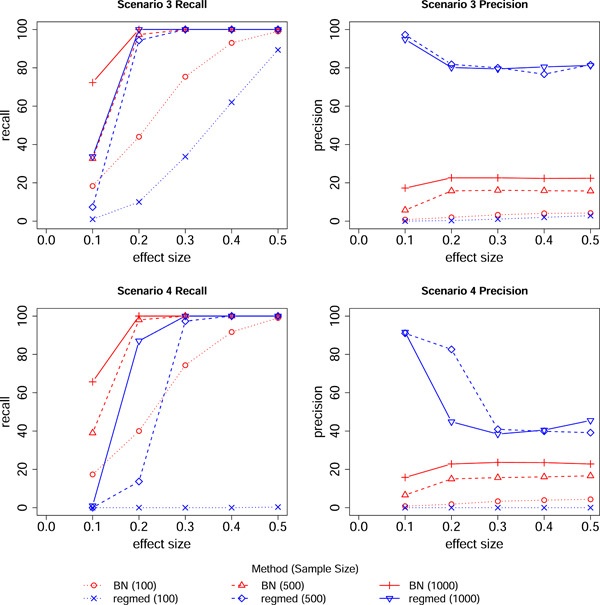
Network recall and precision. Results are for shown for the regmed and Bayesian network (BN) methods for varying values of the effect size for scenarios 3 and 4 and sample sizes of 100, 500, and 1000. Lower covariances were used for these simulations and were set to 0.1 between exposure variables, 0.1 between mediator variables and 0.1 between outcome variables.

These results suggest that regmed is better suited for these type of data sets where there are a large number of variables constrained in these three ordered layers. As the regmed method is specifically designed for this type of analysis (while BayesNetty is designed for a wider scope of analysis), this is perhaps not too surprising. BayesNetty has poor precision due to the large number of variables in the scenarios, particularly exposure variables which may be parent variables to either the mediators or outcome variables. The resulting multiple testing problem causes many incorrect edges to be detected with BayesNetty. To investigate whether this observation might be partly attributable to the high covariances assumed between exposure variables and mediators, we simulated data with lower covariances under scenarios 3 and 4, but did not observe any major differences, see Figure 4.

**Figure 5:**
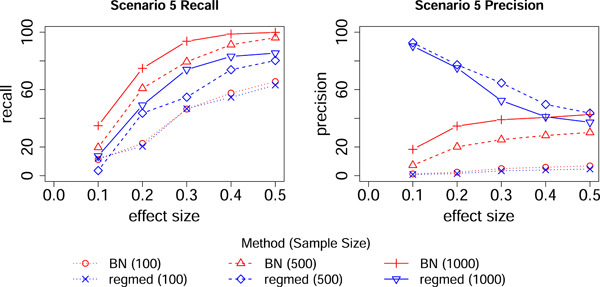
Network recall and precision. Results are for shown for the regmed and Bayesian network (BN) methods for varying values of the effect size for scenario 5 and sample sizes of 100, 500, and 1000. The exposure variables were simulated as SNP data (0, 1, or 2) with a Pearson correlation of 0.5.

A strong motivation for the development of the BayesNetty package was to use genetic factors as “causal anchors,” in an approach closely related to Mendelian Randomization analysis (Howey et al., 2020). Similarly, as demonstrated by Schaid et al. (2022), the regmed package can be employed with genetic variables (SNPs) used as exposure variables. We investigated performance in this situation with data simulated under the model of scenario 5. Results shown are in Figure 5. We see that the results are quite similar to the results when continuous exposures were used (Figure 2, bottom panels), except that the recall and precision seem to converge a bit more slowly as the effect size is increased.

Ideally, we would like to compare the recalls (powers) of BayesNetty, and regmed at the same (fixed) value of the precision (which can be loosely thought of as 1 minus the type I error rate). Unfortunately, this proves complicated, as neither method has a parameter to directly control the false positive rate. Although the penalty parameter λ in regmed and/or the Bayesian Information Criterion (BIC) penalty term used in BayesNetty could—up to a point—be manipulated to try and achieve this purpose, altering these terms fundamentally alters the algorithms proposed, so it is unclear whether the resulting comparison would even be valid. In principle, one could manipulate the recall and precision of BayesNetty through thresholding the identified edges based on their significance (see below). However, in the scenarios considered here, this did not provide sufficient granularity as to generate a useful comparison with regmed.

**Figure 6:**
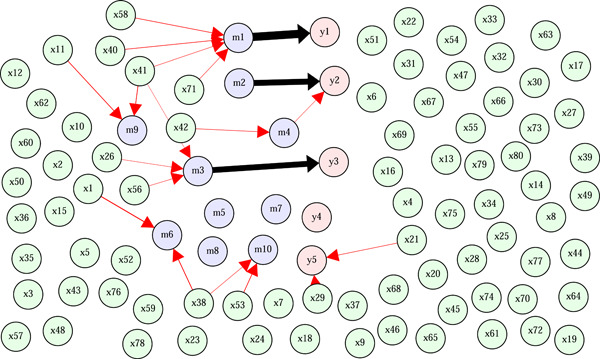
Example best‐fit network from a simulation under scenario 3 with a sample size of 500 and effect size 0.5. The black arrows denote effects that were present in the simulation scenario and red arrows denote effects that were not (i.e., false positives). The thickness of the lines is proportional to the significance of the edges as given by the best‐fit network.

Overall, these results suggest that, to avoid making false positive detections when performing these types of analyses in BayesNetty, the significance of the detected edges should probably be considered, with less confidence given to the weaker edges. In principle, one could make use of BayesNetty's ability to construct average best‐fit networks, through bootstrapping the data many times, to calculate the strength (proportion of times that an edge appears) between any two nodes. A higher threshold, either for significance or strength, can be set to “accept” edges, though this may be somewhat subjective depending on the problem and the desired level of acceptance. A simpler approach is to use the significance of the detected edges in the best‐fit model as an indicator of the strength of evidence. To illustrate this, Figure 6 shows an example best‐fit network for scenario 3 with a sample size of 500 and effect size 0.5. Here it can be seen that the true edges are considerably more significant than the falsely detected edges (note that the results presented here do not attempt to adjust the results for multiple testing). Any “sensible” inclusion threshold would most likely result in the retention of all true edges while discarding most if not all false edges.

We also investigated the running times of the two software packages. As a comparison of typical running times on our system, when there were 1000 individuals with an effect size of 0.5, BayesNetty took 2 min, and 37 s, while regmed took 8 min and 19 s. When there was also missing data with probability 0.25, BayesNetty took 3 min, and 41 s (including imputation time), whereas regmed took only 8 s (due to lack of complete data). However, regmed applied to the imputed data set (as output from BayesNetty) took around 5 min and 42 s.

In conclusion, we find that regmed performs better than BayesNetty for this class of problem with a large number of pre‐specified exposure, mediator, and outcome variables. The results from BayesNetty may need further scrutiny when many variables are present. However, BayesNetty does have the advantage of being a more flexible general method and of having the ability to handle missing data through the use of imputation. The imputation facility of BayesNetty can be used to output a “filled‐in” data set for subsequent analysis by regmed, if desired. Alternatively, one could consider using other imputation/multiple imputation approaches such as multivariate imputation by chained equations (MICE) (Azur et al., 2011). Howey et al. (2021) compared MICE with the approach implemented in BayesNetty (which was specifically tailored to the problem of producing imputed data that would be useful for identifying relationships between variables via subsequent Bayesian Network analysis), and found that BayesNetty generally performed similarly, or slightly better than, MICE, for this purpose.

## AUTHOR CONTRIBUTIONS

Heather J. Cordell conceived and designed the project and played an important role in interpreting the results. Richard Howey carried out data analysis and drafted the manuscript. Both authors contributed to revising the manuscript and approved the final paper.

## CONFLICT OF INTEREST STATEMENT

The authors declare no conflict of interest.

## Data Availability

Simulation scripts/simulated data that support the findings of this study are available from the corresponding author upon reasonable request.
